# A procedure and double-chambered device for macromolecular crystal flash-cooling in different cryogenic liquids

**DOI:** 10.1371/journal.pone.0239706

**Published:** 2020-09-25

**Authors:** Jean-Marc Jeckelmann, Hüseyin Ilgü, Patrick D. Bosshart, Dimitrios Fotiadis

**Affiliations:** Institute of Biochemistry and Molecular Medicine, and Swiss National Centre of Competence in Research (NCCR) TransCure, University of Bern, Bern, Switzerland; University of Queensland, AUSTRALIA

## Abstract

Flash-cooling of macromolecular crystals for X-ray diffraction analysis is usually performed in liquid nitrogen (LN2). Cryogens different than LN2 are used as well for this procedure but are highly underrepresented, e.g., liquid propane and liquid ethane. These two cryogens have significantly higher cooling rates compared with LN2 and may thus be beneficial for flash-cooling of macromolecular crystals. Flash-cooling in liquid propane or liquid ethane results in sample vitrification but is accompanied by solidification of these cryogens, which is not compatible with the robotic systems nowadays used for crystal mounting at most synchrotrons. Here we provide a detailed description of a new double-chambered device and procedure to flash-cool loop mounted macromolecular crystals in different cryogenic liquids. The usage of this device may result in specimens of better crystal- and optical quality in terms of mosaic spread and ice contamination. Furthermore, applying the described procedure with the new double-chambered device provides the possibility to screen for the best flash-cooling cryogen for macromolecular crystals on a routine basis, and, most importantly, the samples obtained allow the usage of state-of-the-art robotic sample-loading systems at synchrotrons.

## Introduction

Structure solution of macromolecules by X-ray crystallography requires the production of 3-dimensional (3D) crystals, which are nowadays almost exclusively analyzed using intense X-ray synchrotron radiation. To reduce radiation damage of the specimen during diffraction experiments, analyses are carried out at cryogenic temperatures, which leads to datasets of better quality [1]. Macromolecular crystals contain 40–80% of solvent. Consequently, and to prevent ice formation, successful crystal flash-cooling protocols include the addition of cryoprotectants such as glycerol or polyethylene glycol (PEG) to the crystallization solution [2]. Therefore, after successful crystal growth and cryo-condition screening [3], the specimen is flash-cooled in a cryogenic liquid, and either directly analyzed or stored at cryogenic temperatures until the diffraction experiment is conducted. The most common cryogenic liquid used for this purpose is liquid nitrogen (LN2), although the flash-cooling protocol itself may play a crucial role to preserve crystal quality [4]. Already in 1982 Silvester and colleagues reported the cooling times of water in various liquid cryogens including LN2, liquid propane and liquid ethane (Table 1) [5]. These results were partially confirmed by Teng and colleagues stating in addition that *i)*. liquid cryogens led to shorter cooling times compared to gaseous cryogens and *ii)*. the shorter cooling time of propane compared to LN2 is a direct consequence of a large difference between their individual boiling points (bp) and melting points (mp) [6]. LN2 cooling is referred to as “film boiling” since due to the small bp-mp difference an insulating film of nitrogen gas is formed around the specimen, resulting in a rather bad heat exchange. In contrast to that, ethane and propane display a large bp-mp difference and thus the dominant cooling process is “nucleate boiling” [6]. However, the correlation between bp-mp difference and cooling rate is not a positive linear function, since ethane has a higher cooling rate than propane but smaller bp-mp difference (Table 1). Nevertheless, “nucleate boiling” turned out to be superior to “film boiling” in terms of cooling rates [6]. If compared to LN2, the higher cooling rates of ethane and propane may have a beneficial effect on crystal quality and could thus lead to structure elucidation at higher resolution. Nevertheless, the number of reported crystal structures that were obtained by flash-cooling macromolecular crystal in LN2 largely exceeds those flash-cooled in liquid propane or liquid ethane. Indeed, a simple Google Scholar (https://scholar.google.com/) search using the search term ["frozen in liquid X" +"crystal structure" -cryo-EM -microscopy] results in more than 60 times more hits for X = nitrogen than X = propane and ethane together. Most likely, the reason for the preferred LN2 usage to flash-cool macromolecular crystals are the flash-cooling protocols applied. For a LN2 based flash-cooling, the specimen is simply plunged in LN2 and then stored in LN2. In comparison, the common procedure for ethane and propane based flash-cooling involves plunging of the harvested crystal sample directly into a LN2 cooled cryo-vial filled with liquid propane or liquid ethane [3]. Like this, the liquid cryogen is allowed to solidify around the vitrified specimen and the sample is then stored in a solid state in LN2 until diffraction analysis. Although such solid samples are transported easier, they are not compatible with the robotic crystal mounting systems used nowadays at synchrotrons. Prior to sample mounting, the solid cryogen has to be partially molten then quickly attached to the goniometer head at the beamline to separate the cryo-vial from the crystal-containing cryo-loop. Potentially remaining solid cryogen is allowed to melt and, if necessary, by short interruptions of the cryo-jet. This time-consuming procedure is done manually and thus requires entering the experimental hutch at the synchrotron for every sample. In addition, this manual procedure might not be reproducible and eventually harmful to the crystal. As a consequence, precious beamtime at the synchrotron is rather used for sample loading than for analysis of crystal diffraction and data collection. Moreover, such a procedure is obviously inapplicable with remote data collection on fully automated beamlines. Therefore, working with such solid samples is inefficient and thus the advantage of the fast and reliable automated crystal sample mounting systems is lost.

**Table 1:** Physical properties of nitrogen, propane and ethane under standard conditions.

Cryogen	Cooling rate^a^ [K^.^s^-1^]	Melting point ^b^ [°C]	Boiling point ^b^ [°C]	Difference ^c^ [°C]
Nitrogen	130	-209.9	-195.8	14.1
Propane	240	-189.7	-42.1	147.6
Ethane	360	-183.3	-88.6	94.7

^a^ Calculated based on experimental cooling curves given in Figs 3 and 6 of reference [5].

^b^ Values were taken from reference [7].

^c^ Boiling point minus melting point.

The flash-cooling time of macromolecular crystal samples depends on several factors [8], e.g., the total volume to be frozen, the crystal size, the type and concentration of the cryoprotectant, and the type of the cryogenic liquid. In order to assess the influence of the latter on crystal quality, ice contamination of the specimen and ultimately the achieved resolution, we here describe an adapted macromolecular flash-cooling procedure based on a newly designed double-chambered device. This device renders the flash-cooling of macromolecular crystals in liquid propane or liquid ethane to a method, which is efficient and compatible with state-of-the-art robotic crystal mounting systems used at synchrotrons. Furthermore, the presented double-chambered device and procedure can be easily and routinely applied in any structural biology laboratory.

## Materials and methods

### Chemicals

Chemicals used to prepare protein purification and crystallization buffers I and II were purchased from Merck in high grades and if possible, in BioUltra grade. For cryogenic liquid generation, gases of high purity were used, i.e., ethane (Carbagas, 99.995%) and propane (Carbagas, >99.95%). Condensation thereof was performed using pure LN2 (Carbagas, 99.8%).

### Preparation of protein crystals

Crystals of the monocarboxylate transporter (SfMCT) from the bacterium *Syntrophobacter fumaroxidans* were obtained as described elsewhere [9, 10]. Briefly, SfMCT was overexpressed in *E*. *coli* BL21(DE3) pLysS after induction with 250 μM isopropyl-β-D-thiogalactopyranoside. The protein was purified from lysed bacteria by differential centrifugation steps, solubilization in *n*-nonyl-β-D-glucopyranoside, followed by nickel affinity chromatography and on-column protease cleavage [11]. Crystals were grown by mixing purified and concentrated SfMCT protein (8 mg/ml) with crystallization buffer I (50 mM HEPES-NaOH pH 7, 5 mM ZnBr2, 30% (v/v) Jeffamine ED-2003) applying the sitting-drop vapor-diffusion method. After 7 days of incubation at 18°C, crystals that grew to comparable size of ~120x50x40 μm were harvested and flash-cooled in various cryogenic liquids by the here described procedure.

For the generation of hen egg-white lysozyme (HEWL) crystals, a solution of HEWL (Merck) at 30 mg/ml in water was mixed in a 1:1 ratio with crystallization buffer II (50 mM Na-acetate pH 4.5, 5% (w/v) NaCl). Applying the sitting-drop vapor-diffusion method, crystals grew after 5–7 days incubation at 18°C as described [12]. Crystals of two different sizes were chosen for the experiments, i.e., larger ones (~150x140x100 μm) and smaller ones (~120x100x80 μm). Prior to flash-cooling, the crystals were soaked in a solution containing 25% (v/v) ethylene glycol, 10% (w/v) NaCl and 100 mM Na-acetate at pH 4.5.

## Results and discussion

**Fig 1:**
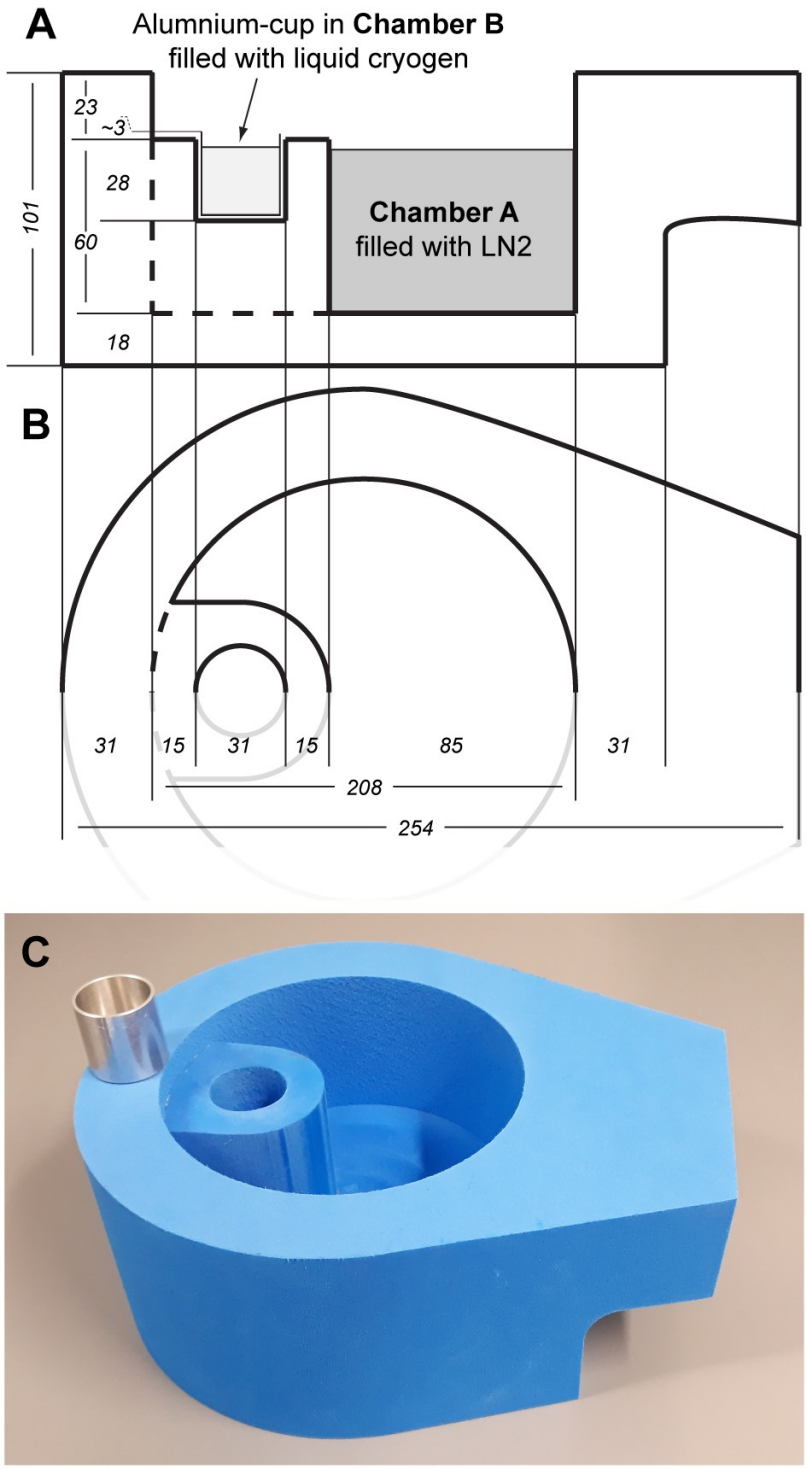
Double-chambered device for flash-cooling macromolecular crystals in different liquid cryogens. (**A** and **B**) Detailed plan of the crystal flash-cooling double-chambered device as viewed from the side (**A**) and the top (**B**). Dimensions are indicated in mm (*italic numbers*). (**C**) Image of the double-chambered device with the aluminum cup placed on top of the device.

Flash-cooling of loop-mounted macromolecular crystals, first in a cryogenic liquid such as propane or ethane, and then second rapidly transfer the vitrified sample into LN2 allows to circumvent the solidification of the cryogen but still makes use of their high cooling rates. Furthermore, these vitrified samples are then stored in LN2 and are thus compatible with state-of-the-art robotic crystal mounting systems used at synchrotrons. In order to realize the described procedure and goal, we have designed a flash-cooling double-chambered device (Fig 1), which was then custom made by Spearlab (https://spearlab.com/).

### Double-chambered flash-cooling device for macromolecular crystals

We designed a double-chambered device (Fig 1), which can be used on a routine basis to flash-cool macromolecular crystals using different cryogenic liquids. The device is based on insulation material and therefore safe to handle. It harbors two compartments, which are physically separated from each other and allow for crystal flash-cooling in two different types of cryogenic liquids, i.e., liquid ethane or liquid propane (Chamber B; Fig 1), and LN2 (Chamber A; Fig 1). Cryogenic liquids such as ethane and propane are not in direct contact with the device but condensed into an aluminum-cup pre-mounted in Chamber B. This aluminum-cup is about 3 mm higher than Chamber B (Fig 1A). This guarantees that i). the device is not harmed due to potential dissolution effects of liquid ethane and liquid propane, and ii). the high thermal conductivity of aluminum is beneficial to maintain the temperature of the cryogen.

### Procedure for flash-cooling of macromolecular crystals in liquid cryogens

The procedure described below is performed in a temperature- and humidity-controlled room, e.g., 18°C and 25% humidity.

#### Prior to flash-cooling of a macromolecular crystal

**Fig 2:**
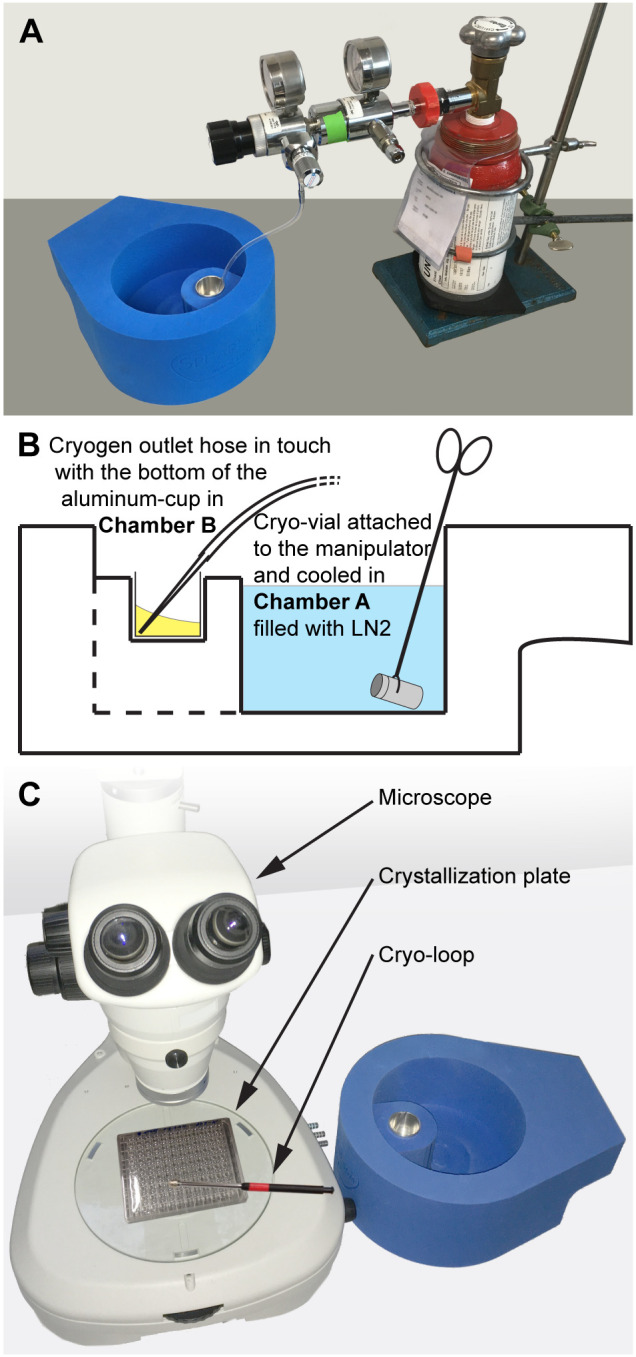
Illustration of the macromolecular flash-cooling procedure. (**A**) Image displaying the cryogen condensation procedure with the double-chambered flash-cooling device placed next to the propane or ethane gas bottle. (**B**) Cartoon describing two different situations, i.e., i). during the cryogen condensation procedure, the gas outlet hose is in contact with the bottom of the aluminum cup in Chamber B (liquid cryogen highlighted in yellow) and ii). after cryogen condensation and prior to crystal harvesting, a cryo-vial is placed into LN2 (highlighted in blue). **(C)** For crystal harvesting, the device is with its Chamber B positioned as close as possible to the microscope to minimize the distance between crystal harvesting site and Chamber B.

The aluminum cup is placed into Chamber B (Figs 1A and 2A) and cooled to cryogenic temperatures by filling it up by two-thirds with LN2. At the same time, Chamber A (Fig 1A) is filled up with LN2 to the level of the aluminum cup (S1 Fig, Step 1). After almost all LN2 has evaporated from the cup (~2 min), the tip of the cryogen’s gas outlet hose is placed into the cup in such a way that the tip is in contact with the bottom (Fig 2A and 2B). The gas valve is opened slowly, and the cryogen gas condensation procedure started. The gas flow rate should be adjusted accordingly to prevent sparkling of the liquid cryogen (S1 Fig, Step 2). Once the aluminum cup is filled up with liquid cryogen, the condensation procedure is stopped. The temperature of the liquid cryogen is measured using a commercially available deep-temperature thermometer and if below -175°C, the system is ready for macromolecular crystal flash-cooling (S1 Fig, Step 3). At this point, it is advisable to slowly place and cool down a first cryo-vial attached to a cryo-vial manipulator in Chamber A (Fig 2B). Finally, the double-chambered flash-cooling device should be positioned such that Chamber B is as close as possible to the site where the crystals are harvested from crystallization drops using cryo-loops, i.e., as close to the microscope as possible (Fig 2C). After a certain time, the liquid cryogen in Chamber B may start to solidify on the surface and on the inner walls of the aluminum cup. Melting frozen cryogen in Chamber B can be achieved by simply placing a dry steel block stored at ambient temperature on the cup for a few seconds. In such a case, temperatures below -175°C should again be verified by measuring prior to the continuation of crystal flash-cooling in liquid cryogens. Usually 5–10 macromolecular crystals can be flash-cooled, then both liquid cryogens should be replaced to avoid contamination with ice, which might have been deposited in the cryogenic liquids due to atmospheric moisture.

#### During flash-cooling of macromolecular crystals

A macromolecular crystal is harvested from the crystallization drop applying the common cryo-loop technique [3] and directly plunged into liquid cryogen (Figs 1 and 2; Chamber B and S1 Fig, Step 3). After 5 seconds of incubation in liquid cryogen, the cryo-loop/crystal is transferred as fast as possible into LN2 (Fig 2B; Chamber A) such that the cold, gaseous N2 layer above the LN2 is not left before plunging the sample into the LN2 (S1 Fig, Step 4). The cryo-loop/crystal is then mounted into the pre-cooled cryo-vial and the vial is further stored in LN2 until X-ray diffraction analysis (S1 Fig, Step 5).

### Flash-cooling of protein crystals

**Fig 3:**
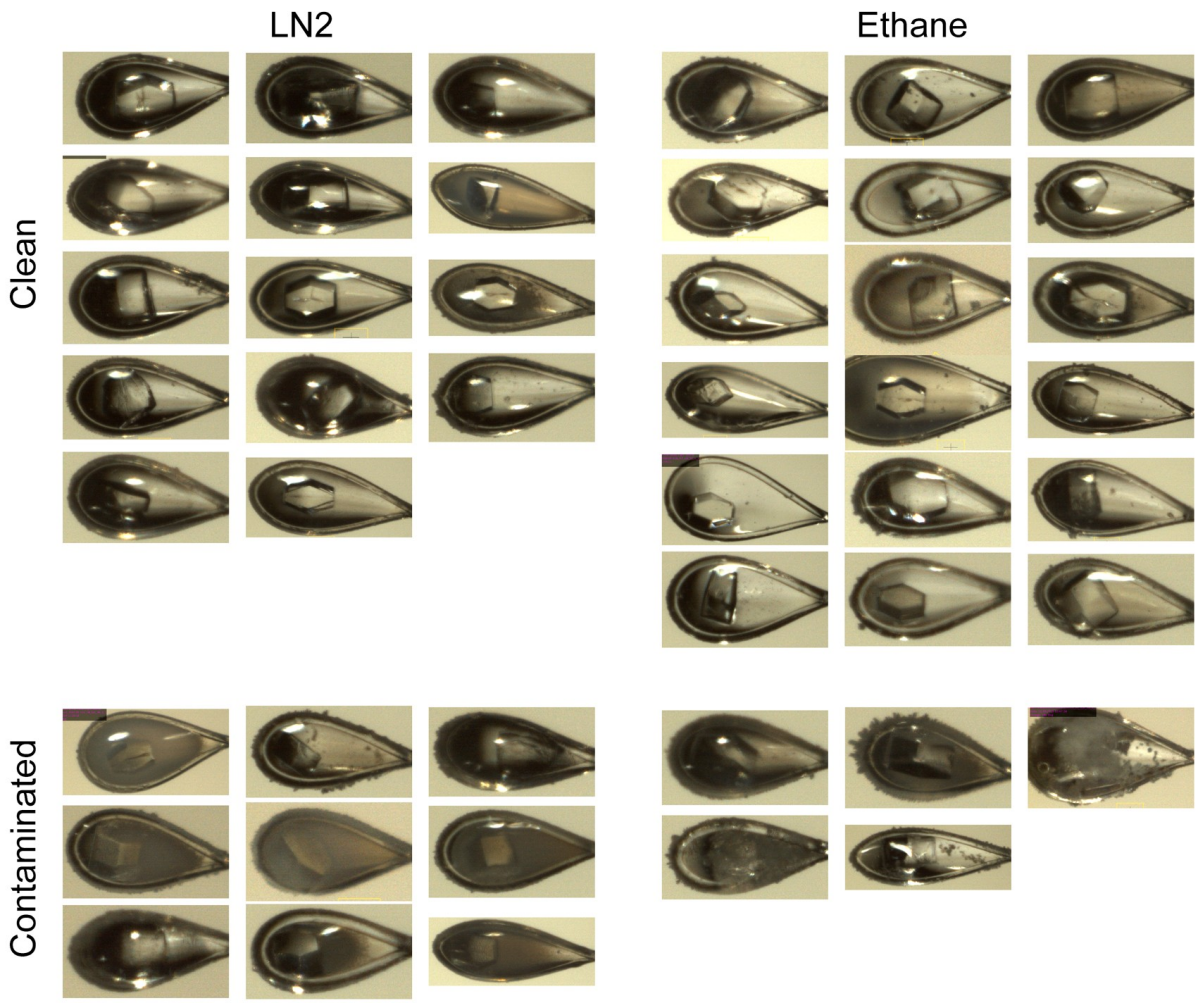
Images of HEWL crystals. Crystals flash-cooled in LN2 (*left*) and ethane (*right*). The top images represent the clean fraction of crystals whereas the images below display samples severely contaminated with ice.

To test the feasibility the flash-cooling procedure using the newly designed double-chambered device and observe potential differences in, e.g., ice contamination of the specimen, crystal quality or diffraction behavior by using different cryogens, two well-characterized proteins were probed, i.e., the soluble protein HEWL and the membrane protein SfMCT. X-ray crystallographic datasets were collected at the Swiss Light Source (SLS) of the Paul Scherrer Institute (PSI) with beam sizes set to 90x50 μm and 60x15 μm for HEWL and SfMCT crystals, respectively. The data was processed using XDS [13] and AIMLESS [14] to predefined resolution cut-offs, i.e., 1.4 Å and 2.8 Å resolution for HEWL and SfMCT crystals, respectively. Based on the unit cell dimensions HEWL and SfMCT crystals are different in solvent content, i.e. ~40% (HEWL) and ~65% (SfMCT) (Table 2). Crystals flash-cooled in propane or ethane were of better optical quality than crystals flash-cooled solely in LN2. Meaning that overall, less ice contaminations were observed on the surface of the loops, e.g., for HEWL crystals about 20% of the mounted loops showed ice contamination if ethane was used as cryogen whereas this amount rose by a factor of two if the crystals were harvested using LN2 only (Fig 3). For comparison, the average mosaicity, and obtained I/σI and CC1/2 values of the highest resolution shell are listed in Table 2. Each group contained five individually harvested, flash-cooled (see previous section) and processed crystals. Whereas SfMCT crystals were of similar size, lysozyme crystals were assigned to two groups depending on their crystal sizes. In terms of crystal quality, the ethane and propane treated crystal showed a trend to slightly lower average mosaic spread than LN2 treated specimens (Table 2). Apart from small variations, there was no obvious difference in I/σI and CC1/2 values between the samples flash-cooled in different cryogenic liquids (Table 2). Larger HEWL crystals were of better quality than smaller ones, but again no clear trend on the cryogen used for flash-cooling could be detected except for the mosaic spread (Table 2).

**Table 2:** Comparison of different cryogenic liquids on crystal quality.

Protein	Approximate Crystal size [μm]	Cryogenic Liquid	Average mosaicity [°]^a^^,^^b^ Mean (Range)	I/σI ^a^^,^^b^ Mean (Range)	CC1/2 [%] ^a^^,^^b^ Mean (Range)
SfMCT^c^	~120x50x40	LN2	0.13 (0.11–0.17)	1.0 (0.4–1.5)	57 (26–75)
	~120x50x40	Propane	0.10 (0.09–0.12)	1.0 (0.8–1.2)	59 (54–64)
	~120x50x40	Ethane	0.09 (0.08–0.10)	1.0 (0.8–1.3)	58 (49–65)
HEWL^d^	~120x100x80	LN2	0.37 (0.29–0.54)	2.7 (1.5–7.8)	81 (59–97)
	~120x100x80	Ethane	0.33 (0.27–0.46)	3.0 (1.6–4.6)	83 (67–93)
HEWL^d^	~150x140x100	LN2	0.34 (0.13–0.61)	5.3 (3.1–8.6)	93 (90–98)
	~150x140x100	Ethane	0.31 (0.25–0.37)	4.7 (4.6–5.1)	94 (93–94)

^a^ Each group contained 5 individually harvested and analyzed crystals. The full data is given in S1 Table.

^b^ The data reflects values of the highest resolution shell, i.e. for HEWL (1.48–1.40 Å) and SfMCT (2.96–2.81 Å).

^c^ Unit-cell: P 4 2 2, a≈79 Å, b≈79 Å, c≈37 Å, α = β = γ = 90°; Solvent content: ~40%.

^d^ Unit-cell: P 2 2 2, a≈61 Å, b≈104 Å, c≈196 Å, α = β = γ = 90°; Solvent content: ~65%.

## Conclusion

Compared to crystals flash-cooled in LN2, liquid ethane or liquid propane treated specimens were in general of better optical quality, i.e., less ice contamination. Furthermore, the crystals showed a slighltly lower mosaic spread, thus reflecting crystals of better quality. The trend of the lower mosaicity of ethane and propane treated crystals, and therefore the slightly better crystal quality was not reflected by I/σI nor CC1/2 values. However, we cannot rule out that in other cases the usage of a specific type of cryogen would be beneficial in terms of diffraction behavior and thus resolution. The flash-cooling procedure presented here using our newly designed double-chambered device (Fig 1) is ideal to check different cryogens on a routine basis. Furthermore, implementing this procedure and double-chambered device into the screening for optimal flash-cooling conditions permits to obtain loop-mounted crystal samples vitrified in cryogens such as ethane and propane without the need to melt the solid cryogen prior to diffraction analysis. This opens the way to screen for the best cryogen and concomitantly use state-of-the-art robotic sample mounting systems at synchrotrons and therefore allows for more efficient crystal sample analysis and beamtime usage.

## Supporting information

S1 Fig Schematic representation of the flash-cooling procedure using the new double-chambered device. On the left-hand side, the flash-cooling Steps 1–5 described in the main text are illustrated and if deemed necessary additional notes are given on the right-hand side.
(PDF)

S1 Table Processing statistics of each individual crystal and data acquisition parameters. (PDF)
