# Supplementary material for: A procedure and double-chambered device for macromolecular crystal flash-cooling in different cryogenic liquids
Source: PLoS One. 2020 Sep 25;15(9):e0239706. doi: 10.1371/journal.pone.0239706 (PMC7518608; doi:10.1371/journal.pone.0239706)
Supplement: S1 Fig — On the left-hand side, the flash-cooling Steps 1–5 described in the main text are illustrated and if deemed necessary additional notes are given on the right-hand side. (PDF) [file pone.0239706.s001.pdf]

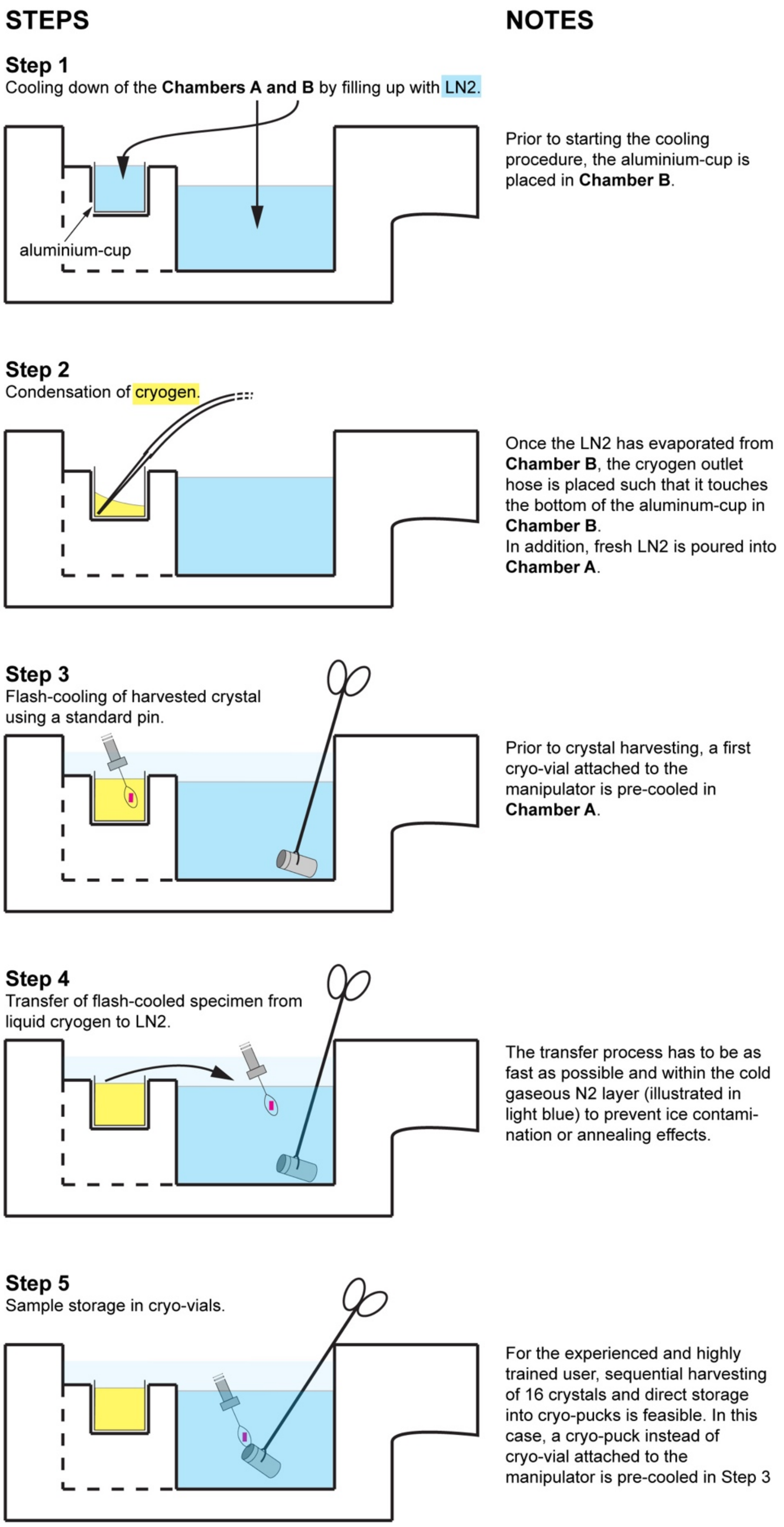

S1 Fig. Schematic representation of the flash-cooling procedure using the new double-chamber device. On the left-hand side, the flash-cooling Steps 1-5 described in the main text are illustrated and if deemed necessary additional notes are given on the right-hand side.
