# Supplementary material for: A procedure and double-chambered device for macromolecular crystal flash-cooling in different cryogenic liquids
Source: PLoS One. 2020 Sep 25;15(9):e0239706. doi: 10.1371/journal.pone.0239706 (PMC7518608; doi:10.1371/journal.pone.0239706)
Supplement: S1 Table — (PDF) [file pone.0239706.s002.pdf]

S1 Table. Processing statistics of each individual crystal and data acquisition parameters.

Cryogen	Protein	Average Mosaicity [°] [a]	I/σI [a]	CC1/2 [%] [a]
		mean	mean	mean
LN2	SfMCT [b]	0.17	0.40	26.2
		0.15	0.80	51.7
		0.12	1.10	66.9
		0.11	1.20	65.1
		0.11	1.50	75.4
		0.13	***1.00***	57.1
Propane	SfMCT [b]	0.09	0.80	54.0
		0.10	0.90	56.5
		0.12	1.00	59.3
		0.11	1.10	58.4
		0.10	1.20	64.2
		0.10	***1.00***	58.5
Ethane	SfMCT [b]	0.09	0.80	53.7
		0.10	0.90	49.2
		0.10	1.00	60.6
		0.08	1.10	64.9
		0.09	1.30	63.1
		0.09	***1.02***	58.3
LN2	HEWL [c] small	0.31	3.70	90.1
		0.34	1.50	58.6
		0.35	1.50	76.3
		0.54	1.30	84.5
		0.29	5.30	97.1
		0.37	***2.66***	81.3
Ethane	HEWL [c] small	0.27	3.80	90.9
		0.46	2.60	83.0
		0.30	2.20	80.8
		0.28	1.60	66.7
		0.35	4.60	93.0
		0.33	***2.96***	82.9
LN2	HEWL [c] Large	0.34	3.70	90.3
		0.15	8.20	98.1
		0.61	2.70	91.1
		0.49	3.10	87.1
		0.13	8.60	98.2
		0.34	***5.26***	93.0
Ethane	HEWL [c] large	0.32	4.40	93.3
		0.31	5.10	94.9
		0.25	4.60	92.5
		0.37	4.90	94.5
		0.30	4.60	93.6
		0.31	***4.72***	93.8

[a] Data reflects values of the highest resolution shell, i.e., for HEWL (1.48-1.40 Å) and SfMCT (2.96-2.81 Å).
[b] Data acquisition parameters: PXI beamline at the Swiss Light Source (SLS) of the Paul Scherrer Institute (PSI); Detector, Dectris Eiger 16M; λ=1 Å; Oscillation angle, 0.2°; Total images recorded 1800.
[c] Data acquisition parameters: PXIII beamline at SLS, PSI; Detector, Dectris Pilatus 2M; λ=1 Å; Oscillation angle, 1.0°; Total images recorded 360.
